# Working collaboratively with people with lived experience to map and co-create guidance on improving health services for people with attention deficit hyperactivity disorder (ADHD)

**DOI:** 10.1192/j.eurpsy.2023.78

**Published:** 2023-07-19

**Authors:** A. Price

**Affiliations:** university of exeter, Exeter, United Kingdom

## Abstract

**Abstract:**

Background: Attention deficit hyperactivity disorder (ADHD) is the most common neurodevelopmental disorder in children and adolescents, with an average worldwide prevalence of 5%. Young people with ADHD experience poorer outcomes than their peers across multiple domains, with treatment shown to reduce these risks. Evidence shows that young people with ADHD can experience multiple challenges when seeking access to healthcare. Debates over how to tackle a ‘failure of healthcare’ for ADHD often include reorganisation of services, including better provision of adult ADHD services, and an expanded role for primary care. However, adult services remain patchy and primary care practitioners feel unsure about how to support young people at this vulnerable stage in their lives, reporting needs for more evidence-based guidance. There is also a lack of national level understanding of the different models, and pathways of care for young people with ADHD aged 16 to 25, hindering efforts to improve access to care and optimise outcomes for this underserved group. Research into this area needs to be guided by people with lived experience of ADHD and informed by perspectives from a range of stakeholders.

Aim: To use collaborative research methods to provide an evidence-base by mapping current services. Then co-produce guidance to improve and better co-ordinate healthcare for young people aged 16-25 with ADHD.

Methods: A national survey about mental health service provision for adults with ADHD (informed by people with lived experience) was developed and distributed. Evidence from this survey stimulated research into primary care service provision, consisting of three interlinked studies guided by research advisory groups of people with lived experience and healthcare professionals

**Disclosure of Interest:**

None Declared

